# Protocol for AI-supported immunofluorescence colocalization analysis in human enteric neurons

**DOI:** 10.1016/j.xpro.2025.103828

**Published:** 2025-05-16

**Authors:** Egan L. Choi, Yuebo Zhang, Fei Gao, Nick M. Huynh, Alejandro Stark Quiroz, Adil E. Bharucha, Yujiro Hayashi, Tamas Ordog

**Affiliations:** 1 Enteric Neuroscience Program and Department of Physiology and Biomedical Engineering, Mayo Clinic College of Medicine and Science, Rochester, MN 55905, USA; 2 Gastroenterology Research Unit, Division of Gastroenterology and Hepatology, Department of Medicine, Mayo Clinic College of Medicine and Science, Rochester, MN 55905, USA; 3 Division of Gastroenterology and Hepatology, Department of Medicine, Mayo Clinic College of Medicine and Science, Rochester, MN 55905, USA

**Keywords:** Microscopy, Molecular Biology, Neuroscience

## Abstract

Gastrointestinal motor functions are orchestrated by enteric neurons that display a high degree of anatomical and functional variability. Here, we present a protocol for AI-supported immunofluorescence colocalization analysis in human enteric neurons. We describe steps for human gastric tunica muscularis preparation, immunofluorescent labeling of enteric neurons, and quantification of marker colocalization using Nikon NIS-Elements. This protocol addresses the challenges presented by the high density of neurons within enteric ganglia, overlapping fluorescence signals, background fluorescence, and potential user bias in quantification.

For complete details on the use and execution of this protocol, please refer to Gao et al.^1^

## Highlights

• Protocols for the preparation of human gastric tissues and cryosection immunostaining
• Immunofluorescence confocal microscopy for the detection and visualization of enteric neurons
• Quantitative image analysis for colocalization by artificial intelligence (AI)

## Abstract (editor highlights)

Publisher’s note: Undertaking any experimental protocol requires adherence to local institutional guidelines for laboratory safety and ethics.

## Graphical abstract

**Figure:**
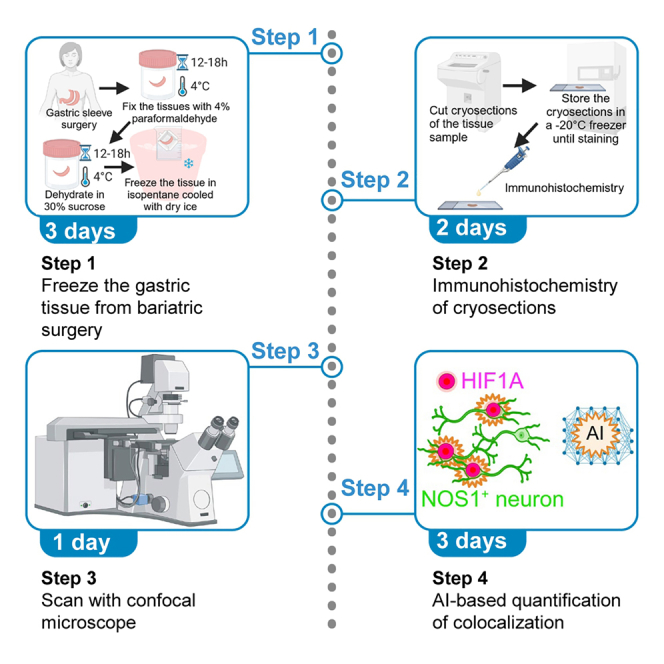

## Before you begin

Nitric oxide, a gaseous neurotransmitter produced by neuronal nitric oxide synthase 1 (NOS1) in a subset of neurons of the enteric nervous system, is critical for normal gastrointestinal motility through the regulation of smooth muscle contractions, peristalsis, and reflexive movements.^2^ We have recently discovered that *NOS1* transcription is controlled by hypoxia-inducible factor 1 alpha (HIF1A),^1^ and that reduced HIF1A levels lead to the downregulation of NOS1, an important factor of diabetic gastroparesis.^1^^,^^2^^,^^3^ Therefore, assessing the expression levels of HIF1A and NOS1 in enteric neurons is crucial for understanding the roles of these proteins in the pathogenesis of diabetic gastroparesis and other gastrointestinal neuromuscular disorders. However, accurate determination of protein colocalization in immunohistochemistry applications is challenged by spectral overlap and autofluorescence causing false signals, imaging artifacts affecting accuracy, and imbalanced protein expression obscuring detection.^4^ To address these challenges, we have developed a new protocol to improve quantification of target proteins in enteric neurons using artificial intelligence (AI)-based image analysis.^1^ The method involves preparing the human gastric tunica muscularis, immunofluorescent labeling of targets, and performing detailed quantification of colocalization using Nikon NIS-Elements, an AI-powered imaging software for advanced microscopy analysis.^5^^,^^6^ Our approach enables a more precise, 3-dimensional (3D) quantitative analysis of protein colocalization in enteric neurons by confocal microscopy than allowed by conventional methods, which are limited by overlapping fluorescence signals, autofluorescence,^7^ and potential user bias in quantification.

### Institutional permissions

This study was approved by the Mayo Clinic Institutional Review Board (IRB) (protocol numbers 13-008138 and 20-012002). A total of 3 non-diabetic female participants (58.33 ± 7.36 years old), 3 non-diabetic male participants (45 ± 4.85 years old), and 7 female diabetic participants (47.86 ± 8.30 years old) were included in the study. Detailed patient information is provided in Table 1.

**Table 1:** Participant demographics by age, sex, diabetes status, and IRB approval number

Age	Sex	DM or non-DM	IRB
52	F	non-DM	13–008138
47	F	DM	13–008138
54	F	DM	13–008138
40	F	non-DM	13–008138
40	F	DM	13–008138
42	F	DM	13–008138
47	F	non-DM	13–008138
67	M	non-DM	20–012002
41	F	non-DM	20–012002
49	M	non-DM	20–012002
59	M	non-DM	20–012002
36	F	DM	20–012002
59	F	DM	20–012002

This table presents the age, sex, diabetes status (DM, diabetes mellitus; non-DM, non-diabetic mellitus), and IRB approval numbers for each study participant.

## Key resources table

**Table:** 

REAGENT or RESOURCE	SOURCE	IDENTIFIER
**Antibodies**		

HIF1A (1.66 μg/mL; 1:300 dilution)	Thermo Fischer	Cat#; #700505 Lot#; 2650893 RRID: AB_2532327
NOS1 (0.66 μg/mL; 1:300 dilution)	Santa Cruz Biotechnology	Cat#; sc-5302 Lot#; C0817 RRID: AB_628099
Secondary Ab: Alexa 647 anti-rabbit IgG (4.44 μg/mL; 1:450 dilution)	Life Technologies	Cat#; A31573 Lot#; 2577247 RRID: AB_2536183
Secondary Ab: Alexa 488 anti-mouse IgG (4.44 μg/mL; 1:450 dilution)	Life Technologies	Cat#; A21202 Lot#; 1890861 RRID: AB_141607

**Chemicals, peptides, and recombinant proteins**		

2-Methylbutane	Sigma-Aldrich	320404-1L
DAPI (4′,6-diamidino-2-phenylindole, dilactate)	Thermo Fisher Scientific	D3571
F-12 Nutrient Mixture	Gibco	21700–075
Lens cleaning solution 1OZ spray bottle	Nikon	77013066
Microscope slides	CardinalHealth	M6133A
Nail polish	SINFUL COLORS	733854978163
Normal donkey serum	Jackson ImmunoResearch	017-000-121 Lot#: 158760
Paraformaldehyde	Sigma-Aldrich	158127-500G
SlowFade Diamond antifade mountant (mounting media)	Thermo Fisher Scientific	S36972
Sodium bicarbonate	Sigma-Aldrich	900506-1KG
StainTray 10 slides staining system	Simport Scientific	M918-2
Sucrose	Sigma-Aldrich	S9378-1KG
Surface-Amps X-100 (Triton X)	Thermo Fisher Scientific	28314
Sylgard184	Electron Microscopy Science	24236–10
VECTASHIELD HardSet Antifade Mounting Medium	Vector Laboratories	H-1400-10

**Software and algorithms**		

BANDICAM	Bandicam Company	https://www.bandicam.com
Microsoft Excel	Microsoft	https://www.microsoft.com/en-us/microsoft-365/excel
NIS Elements Version 6.02.01	Nikon	https://www.microscope.healthcare.nikon.com/products/software/nis-elements/nis-elements-advanced-research
NIS-Elements AR 6.02.01	Nikon	https://www.nisoftware.net/NikonSaleApplication/Help/Docs-AR/eng_ar/nis.ai.html

**Other**		

15 mL polypropylene conical tube	Falcon	352097
Barnstead Nanopure Diamond System	Fisher Scientific	D11931
BD Needle 1 1/2 in. single use, sterile, 20 G	BD	305176
Fisherbrand Disposable base molds	Fisher Scientific	22-363-556
Dumont AA - Epoxy-coated forceps	Fine Science Tools	11210-10
Falcon 100 mm x 15 mm Not TC-treated bacteriological Petri dish, 20/pack, 500/case, sterile	Falcon	351029
FLEX TUBE 1.5 mL, natural	Eppendorf	022364111
High-profile disposable blades 818	Leica	14035838383
ImmEdge Pen	Vector Laboratories	H-4000
LEICA cryostat	Leica	CM1950
Simport Scientific StainTray Slide Staining System	Simport	22-045-035
Surgical scissors - large loops	Fine Science Tools	14101–14
Tissue-Tek O.C.T. Compound	Sakura Finetek	4583
Kimwipes	Kimtech	34120
Microscope slides	CardinalHealth	M6133A
Microscope cover glass	Fisher Scientific	12541016
Rapid-Flow Sterile disposable filter units with PES	Thermo Fisher Scientific	568–0020
Rotomix 50800 Orbital shaker	Thermolyne	50800

## Step-by-step method details

### Preparing cryosections of human gastric muscles

**Timing: 3 days**

This section outlines how to prepare cryosections from human gastric muscle, including fixation, dehydration, embedding, and cryostat sectioning for immunofluorescence.

1. After gastric sleeve surgery is completed, collect human stomach specimens.

***Note:*** These tissues are regarded as waste material resulting from gastric sleeve surgery for obesity treatment.

**Figure 1:**
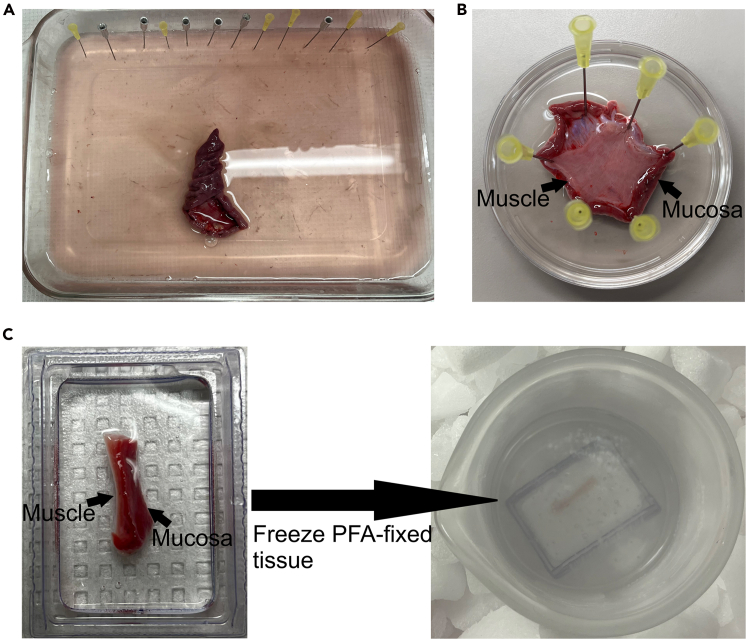
Preparation of human gastric tissue for cryosectioning (A) Photo of gastric tissue obtained following gastric sleeve surgery. (B) Gastric tissue fixed with 20G needles in 4% paraformaldehyde (PFA). (C) Step-by-step images demonstrating the freezing of gastric tissue in OCT compound using cold isopentane on dry ice.

2. Keep stomach in cold F12 medium and transport it to the lab, without delay, in a bag with ice packs to minimize ischemic time and to ensure the tissue remains viable.
3. Place the stomach in a silicone-based tray (Figure 1A).
4. Stretch muscle using 20G needles in silicone-based dish just enough to keep it flat but avoid applying excessive tension (e.g., >120% of unstretched dimensions) to preserve tissue integrity (Figure 1B).
5. Fix the tissues with 4% paraformaldehyde (PFA) for 12-18 h at 4°C.

***Note:*** Preparation of 4% PFA is described in Table 2.

6. Wash with cold phosphate-buffered saline (PBS) on a rocker for 1 h, changing to fresh PBS at 10 min, 20 min, and 30 min.

***Note:*** Preparation of PBS is described in Table 3.

7. Dehydrate in 30% sucrose prepared in PBS on a rocker for an additional 12-18 h at 4°C.
8. Place tissues into Tissue-Tek O.C.T. compound in Fisherbrand disposable base molds, then freeze them in isopropanol on dry ice (Figure 1C).
9. Cut frozen tissues into 12 μm sections using a cryostat.

***Note:*** Tissues should be processed as soon as possible to ensure the viability of the gastric muscle.

**Table 2:** Preparation of 4% paraformaldehyde solution

4% PFA solution		
Reagent	Final concentration	Amount
PBS	1×	200 mL
Paraformaldehyde	4%	8 g
Total	N/A	200 mL

Stir the 4% PFA solution with heat for at least 30 min until fully dissolved in PBS. Then, filter the solution using Rapid-Flow Sterile Disposable Filter Units with PES. Store at 4°C for up to 1 week.

**Table 3:** Preparation of 10× phosphate-buffer saline composition

10× PBS solution composition		
Reagent	Source	Amount for 1L
Sodium phosphate dibasic heptahydrate	SIGMA-ALDRICH S9390	20.1 g
Sodium phosphate monobasic dihydrate	SIGMA-ALDRICH 04269	3.9 g
Sodium chloride	SIGMA-ALDRICH S9625	84.7 g

Dissolve the reagents in Milli-Q lab-grade water with stirring for at least 30 min until fully dissolved. To prepare 1× PBS, dilute 100 mL of 10× PBS with 900 mL of Milli-Q lab pure water.

### Staining of NOS1 and HIF1A for quantification

**Timing: 2 days**

This section describes immunostaining for NOS1 and HIF1A in gastric cryosections to enable fluorescence-based quantification.

10. Create a water-repellent barrier around the tissues on the microscope slide using the ImmEdge Pen.
11. Wash the tissues with cold PBS for 5 min to dissolve the O.C.T. compound.
12. Permeabilize the tissues using 0.25% Triton X-100 prepared in PBS for 50 min at room temperature (RT; 21-23°C).
13. Incubate the tissues with 10% normal donkey serum prepared in PBS for 1 h RT (21-23°C) to block non-specific staining.
14. Incubate primary antibodies hypoxia-inducible factor 1 alpha (HIF1A, 1.66 μg/mL; 1:300 dilution) and nitric oxide synthase 1 (NOS1, 0.66 μg/mL; 1:300 dilution) with cold 5% donkey serum containing 0.5% Triton X for 12 h at 4°C.

***Note:*** Donkey serum can be stored at 4°C for at least 3 months. To ensure consistent staining, prepare a 5% donkey serum solution fresh each time.

15. After removing the primary antibodies using suction, wash with PBS for 15 min at RT (21-23°C).
16. Incubate secondary antibodies (Alexa Fluor 647 anti-rabbit IgG and Alexa Fluor 488 anti-mouse IgG both at 4.44 μg/mL; 1:450 dilution) with cold 5% donkey serum containing 0.5% Triton X for 1 h at RT (21-23°C).
17. After gently aspirating the primary antibodies solution, wash with PBS for 15 min at RT (21-23°C).
18. Stain with DAPI (4′,6-diamidino-2-phenylindole) at 1:300 dilution for 1 h at RT (21-23°C).

***Note:*** Compared to other cell types, neurons exhibit weaker nuclear staining with DAPI. Prolonged incubation for at least 1 h improves staining intensity. Detailed information about the antibodies is provided in the key resources table.

19. Rinse once with ultrapure water to remove any remaining salt.
20. Mount sections with VECTASHIELD HardSet Antifade Mounting Medium.
21. Seal the coverslip using non-fluorescent lacquer nail polish.

### Imaging enteric neurons using confocal microscope

**Timing: 1 day**

This section explains how to acquire z stack confocal images using Nikon NIS-Elements for 3D visualization and quantification.

**Figure 2:**
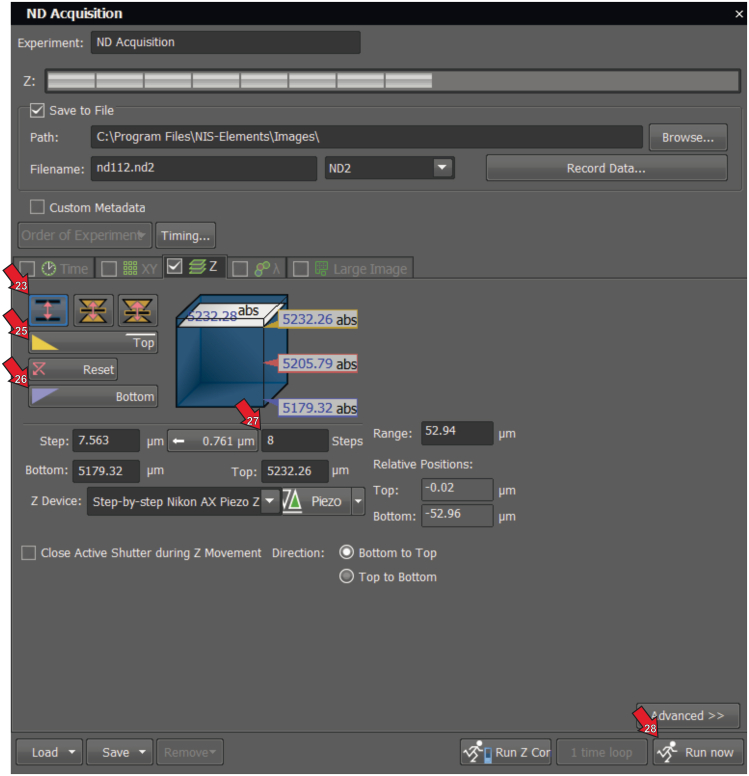
Screenshot of the ND Acquisition window showing z stack parameter settings

22. Acquire a 3D image using the Z-series Acquisition on Nikon Elements software. The workflow for ND acquisition is illustrated in detail (Figure 2).
23. Select the Acquisition tab and click the z stack option.
24. Define the Z range by specifying the top and bottom positions of the gastric muscle.
25. Focus on the top of the muscle and click the “Top” button.
26. Move the focus to the bottom of the muscle and press the “Bottom” button.
27. Set 8 steps to cover the entire depth of the muscle for accurate quantification.
28. Click “Run Now” to begin the acquisition. Save the image as an ND file (Figure 2).

**Figure 3:**
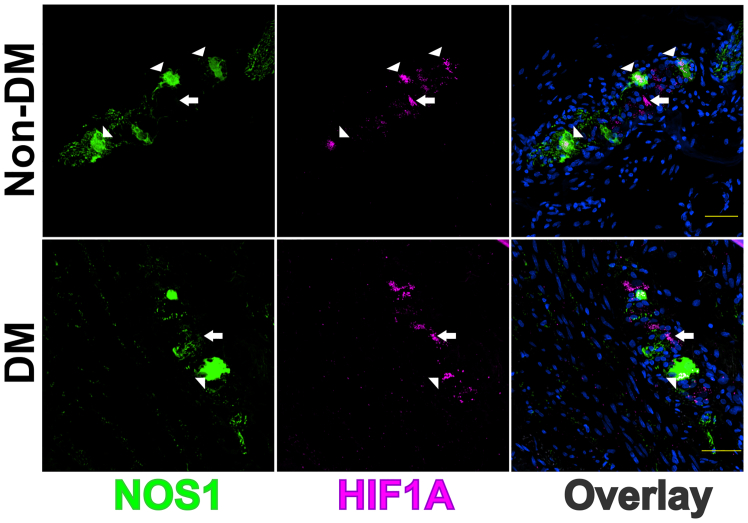
Confocal images of cryosection from non-diabetic (Non-DM) and DM patients *Top panels*: Representative confocal images of cryosections from a patient without diabetes showing predominantly nuclear HIF1A in NOS1^+^ (arrowhead) and NOS1^−^ (arrow) neurons. *Bottom panels*: Representative confocal images of cryosections from a patient with diabetes mellitus (DM) showing reduced HIF1A in NOS1^+^ (arrowhead) and NOS1^−^ (arrow) neurons. Scale bar: 50 μm.

***Note:*** Representative images of NOS1 and HIF1A in the gastric muscles of human patients with and without diabetes are presented in Figure 3. As shown previously,^3^^,^^8^ NOS1 positive enteric neurons are reduced in diabetic patients. Set exposure parameters in NIS-Elements to prevent pixel values from saturating and to avoid loss of detail in bright regions for accurate NOS1 and HIF1A colocalization. Detailed image acquisition parameters of Nikon AX R confocal system are provided in Table 4.

**Table 4:** Image acquisition parameters of Nikon AX R confocal system

Image acquisition parameters	
Acquisition setup	Settings for whole mount staining
Acquisition Mode	Laser scanning confocal
Scan Mechanism	Galvano
405 nm (DAPI)	Emission 450
Laser Power	2.9
Gain	66.9
488 nm (eGFP)	Emission 525
Laser Power	19.3
Gain	7.2
640 nm (AF647)	Emission 700
Laser Power	18.9
Gain	7.2
Pixels	1024 × 1024

### Immunohistochemistry analysis and quantification

**Timing: 5 min**

This section focuses on AI-supported segmentation and quantification of NOS1 and HIF1A using Nikon’s GA3 in NIS-Elements to generate reliable, quantitative fluorescence data.

**Figure 4:**
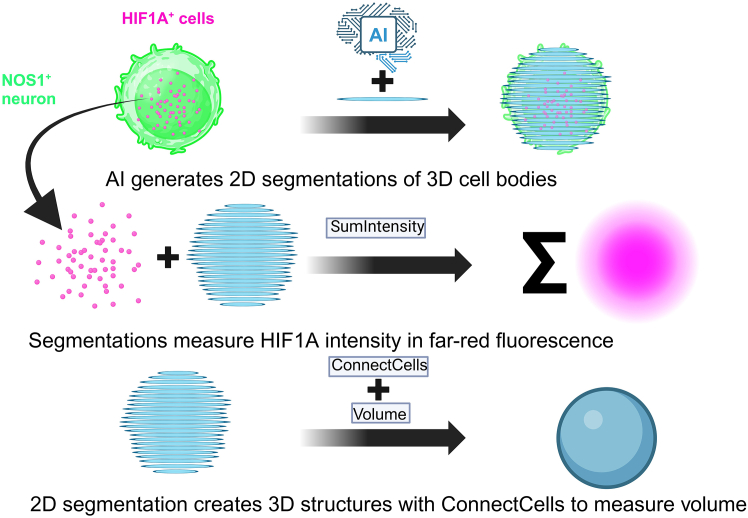
Conceptual visualization of AI-based segmentation and quantification A visual summary of the image analysis pipeline, illustrating the steps of AI-driven segmentation and the processing of quantification results. The figure highlights the workflow from initial image input to the generation of final quantified data.

This step focuses on the segmentation and quantification of NOS1 variables, such as volume, using Nikon’s NIS Elements software. The process involves adjusting frame brightness and contrast levels, as well as performing background subtraction. These steps, along with segmentation and volume extraction, are executed using Nikon’s general analysis 3 (GA3), a modular interface for image analysis. In GA3, analysis workflows are visually represented as graphs, known as “recipes,” which are composed of individual steps called “nodes.” Each node is connected to others through labeled attachments, facilitating stepwise image analysis. Figure 4 provides a conceptual overview of this AI-powered image analysis pipeline. It illustrates the segmentation of enteric neurons and the downstream quantification processes, highlighting how input images are processed to generate precise, quantitative data including the sum fluorescent intensities of HIF1A puncta and the volume of NOS1^+^ enteric neurons for further analysis.

***Note:*** Accurate segmentation is crucial for reliable measurements. The use of AI-based tools minimizes user bias and manual errors while improving the consistency and precision of image analysis. Proper calibration of the software and careful handling of the imaging process are essential for obtaining high-quality data.

### AI training for neuron segmentation

**Timing: 3 days**

This step focuses on the segmentation and quantification of NOS1 variables, such as volume, using Nikon’s NIS Elements software.

**Figure 5:**
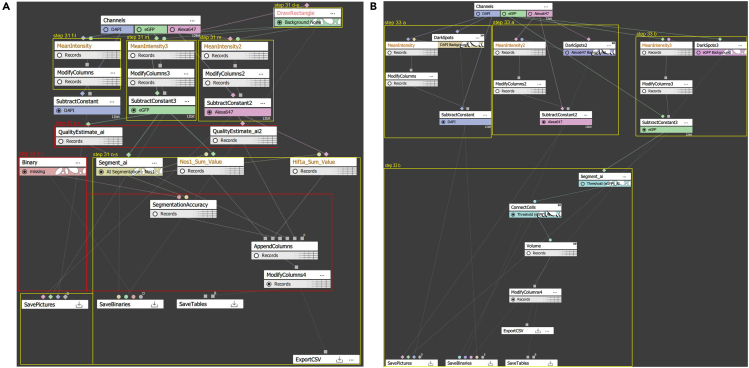
Quantitative image analysis workflow diagram (A) GA3 recipe for obtaining cumulative fluorescence intensities within flattened segmented areas of nitrergic neurons using Nikon’s Segment.ai algorithm. (B) GA3 recipe for obtaining the volumes of segmented areas across optical sections containing the entire nitrergic perikarya using Nikon’s Segment.ai algorithm. Yellow and red lines correspond to specific steps detailed in the main text. This Figure 5 is a modified version of supplemental Figure 3 from our recent publication^1^ and is reproduced here with permission from the Elsevier Publishing Group.

This step enables the training of AI models to achieve highly accurate neuron segmentation. The workflow for neuron segmentation and the AI training process is illustrated in Figure 5 and summarized in Table 5 to provide a structured overview.

**Figure 6:**
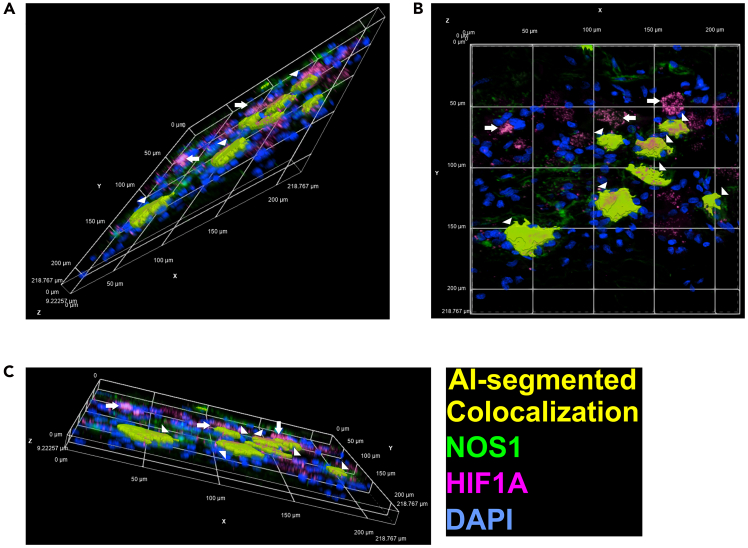
Confocal images of cryosections from a non-diabetic DM patient (3D reconstruction) Confocal images of cryosections from a non-diabetic DM patient (3D reconstruction). Representative confocal images of cryosections showing predominantly nuclear localization of HIF1A in NOS1^+^ (arrowhead) and NOS1^−^ (arrow) neurons. Yellow color indicates the colocalization of HIF1A and NOS1^+^ neurons. Panels (A, B, and C) represent 3D reconstructions of the same region viewed from different angles to highlight the spatial relationship and localization of HIF1A within the neurons.

29. Manual neuron segmentation:
  a. Open a high-quality image of enteric neurons in NIS Elements, ensuring sufficient signal-to-noise ratio, contrast, and sharpness for accurate AI-based segmentation.
  b. Use the Threshold tool in NIS Elements to segment the enteric neurons.
  c. Use the segmentation drawing tool to erase any incorrect non-neuronal cell segmentations.
  d. Repeat steps b and c for five or more high-quality images (see Figures 6A–6C for final 3D segmentation examples).

***Note:*** A sufficient signal-to-noise ratio (SNR) means that the fluorescence intensity of the target signal is clearly distinguishable from background noise, such as autofluorescence, detector noise, or non-specific staining. Five images are sufficient for the training of the AI software. Fewer images will lead to a higher chance of imperfect segmentation.

**Figure 7:**
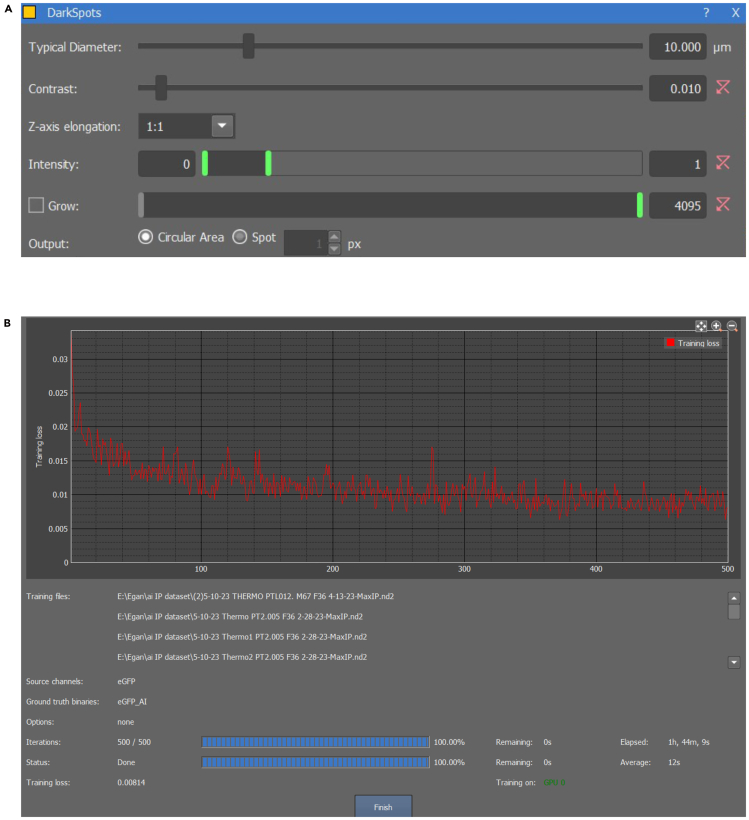
Examples of various components in the NIS-Elements AI segmentation workflow (A) DarkSpots showing typical diameter, Contrast, Z-axis elongation, intensity and grow. (B) Training loss graph showing output after 2000 iterations of AI training of colocalization HIF1A and NOS1^+^ neurons.

**Figure 8:**
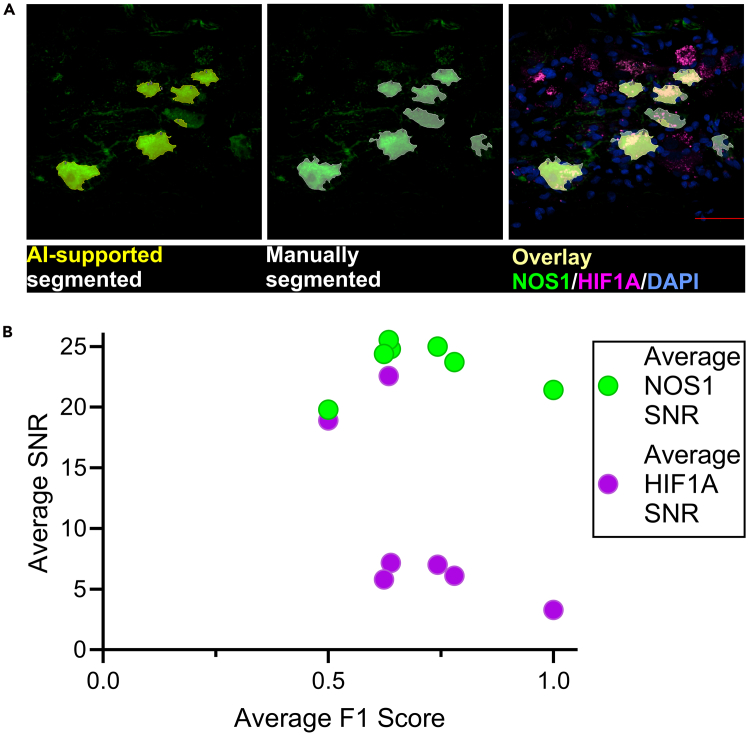
Validation of AI-supported segmentation and quantitative analysis (A) Confocal images of 3D-reconstructed cryosections from a non-diabetic gastroparesis patient. Regions outlined in yellow are AI-segmented areas of NOS1^+^ immunofluorescence. Regions outlined in white represent manually segmented NOS1^+^ neurons. Scale bar: 50 μm. (B) Quantitative analysis of segmentation accuracy and signal-to-noise ratio (SNR) for NOS1 and HIF1A staining. Each dot represents a z stack image (n = 7), with green and magenta circles corresponding to average SNR values for NOS1 and HIF1A, respectively. F1 scores (x-axis) were calculated using the default Nikon intersection-over-union (IoU) threshold of 0.5 and plotted against the corresponding average SNR (y-axis).

30. AI training:
  a. Use the previously segmented images to train a Segment.ai model for accurate replication of neuron segmentation.
  b. Open the Segment.ai training program. Select the previously prepared training images.
  c. Choose the source channel for NOS1 and the ground truth binary used to segment the NOS1^+^ neurons.
  d. Set the number of iterations for the model to train as 500.
  e. Save the model with an appropriate designation, such as NOS1-AI.
    ***Note:*** Perform at least 500 training iterations to minimize error in the AI software to an optimal level. Fewer iterations will reduce training time but increase the risk of errors due to higher training loss. The training process may take between 1.5-6 h or longer, depending on the number of iterations.
    i. Evaluate the accuracy of the AI software quantitatively based on the training loss graph (see Figure 7A as an example) and qualitatively by applying it to unsegmented images.
    ii. Visually check the AI software by applying it to non-training set images.
    iii. Retrain the model if there are any visually apparent false positive or false negative segmentations.
    iv. Retrain the model as needed until the segmentation accuracy approaches optimal range (0.01–0.1).
      ***Note:*** While achieving zero training loss is unattainable, an ideal range is between 0.01 and 0.1. Higher values may result in increased false positives or false negatives. Use the training loss graph output by the system as a key metric to measure accuracy. Training loss is a common measure in deep learning programs, indicating how accurately the AI software replicates the segmentation of the training images. Lower training loss corresponds to greater accuracy. Make sure by visual inspection there are no false positives or false negatives when applying the model to non-training set images. False positives occur when NOS1-positive cells without neuron morphology are segmented. False negatives occur when NOS1-positive cells with neuron morphology are not segmented.
31. AI-supported neuron sum fluorescence analysis:
  a. Use the built-in GA3 recipe system to create a new GA3 recipe (see Figure 5A for the expected final recipe layout).
  b. Apply background reduction using the nodes DrawRectangle, MeanIntensity, ModifyColumns, and SubtractConstant.
  c. Use the search tab in the top-right corner of the GA3 recipe window to locate these nodes and drag one of each onto the programming window.
    ***Note:*** Use DrawRectangle to select an area of the image where background is detected (ensure the rectangle does not overlap stained cells). Use MeanIntensity to average the intensity of each channel. Apply ModifyColumns to isolate the mean intensity value by removing unnecessary data columns. Use SubtractConstant to reduce the channel’s fluorescence using the mean intensity value as a constant.
  d. Click and drag the 'A' module of the DrawRectangle node to one of the channels.
  e. Draw a rectangle on the images where no enteric neurons or DAPI-stained nucleus are located.
    ***Note:*** This creates a segmentation layer that appears as a colored node in GA3.
  f. Click and drag the 'A' module of the MeanIntensity node to the DAPI channel.
  g. Then, click and drag the 'B' module to the colored node created by DrawRectangle.
  h. Click and drag the 'A' module of the ModifyColumns node to the Records node connected to MeanIntensity.
  i. Open the drop-down menu in ModifyColumns and uncheck the box next to 'ZStackIndex' (see Figure 5A).
  j. Click and drag the 'A' module of the SubtractConstant node to the DAPI channel.
  k. Right-click on SubtractConstant and enable the P0 variable to create a P0 module connection.
  l. Attach the P0 module to the Records node connected to ModifyColumns.
    ***Note:*** This will create a new DAPI binary layer.
  m. Repeat steps g through l for each channel (HIF1A and NOS1).
  n. Apply segmentation and sum fluorescence values using the nodes Segment.ai, SumIntensity, and JoinRecords.
    ***Note:*** Segment.ai node allows you to include any AI you created into the GA3 workflow, SumIntensity creates a cumulative value from all fluorescent value within a given segmentation, and JoinRecords combines two different tables.
  o. Create a Segment.ai node and attach it to the new NOS1 binary layer.
  p. Click the drop-down menu and select the AI software you created previously.
  q. Create two SumIntensity nodes and attach the “A” module of each to the binary layer node created by the Segment.ai node.
  r. Attach the “B” module of one node to the NOS1 channel and attach the other to the HIF1A channel.
  s. Label appropriately (Hif1a_Sum_Value).
32. Quantitative evaluation of AI segmentation performance using GA3:
  a. Locate ‘QualityEstimate_ai’ node in the GA3 recipe window, and connect it to the Nos1 binary layer and Hif1a binary layer created after ‘SubtractConstant’.
    ***Note:*** QualityEstimate_ai returns the estimated signal to noise ratio (SNR) value of the binary layers it is connected to.
  b. To objectively quantify the metrics of performance of the AI model compared to manual segmentation of enteric neurons, manually segment according to steps 29b and c. Then store the binary and label it as ‘Manual Segmentation-Nos1’.
  c. To objectively quantify the metrics of performance of the AI model compared to manual segmentation of enteric neurons, manually segment according to steps 29b and c. Then store the binary and label it as ‘Manual Segmentation-Nos1’.
    ***Note:*** Comparison of AI-supported and manual segmentation of NOS1^+^ neurons is shown in Figure 8A and Methods Video 2.
  d. Use the search tab in the top-right corner of the GA3 recipe window to locate ‘SegmentationAccuracy’.
    ***Note:*** SegmentationAccuracy calculates the average precision to evaluate AI performance in object segmentation. It takes two inputs, Ground Truth (GT) and Prediction (Pred), and compares the ground truth binary layer (A) with the predicted binary layer (B) generated by the AI model. Objects from both layers are paired and classified based on the intersection over Union (IoU) threshold into true positives (TP), false positives (FP), and false negatives (FN). True positives represent correctly matched objects, false positives indicate incorrectly segmented objects, and false negatives represent missed detections. Using these classifications, the node calculates key performance metrics: precision (TP / [TP + FP]), recall (TP / [TP + FN]), and the F1 score (2 × precision × recall / [precision + recall]). The IoU threshold defines the minimum overlap required for two objects to be considered a correct match. The default IoU threshold is 0.5, meaning a predicted object must overlap with a ground truth object by at least 50% to be considered a true positive.
  e. Locate ‘Binaries’ under ‘Source Binary’ and move to the GA3 recipe.
  f. Upload the stored manually segmented binary to the ‘Binary’ node under ‘Name’.
  g. Connect ground truth (GT) module to the ‘Binary’ node and the predicted (Pred) module to the Segment_ai’ node.
  h. Create an “AppendColumns” node and attach it to both Records nodes created by the two SumIntensity nodes, the QualityEstimate_ai nodes, and the SegmentationAccuracy node. Record the resulting table using an Excel sheet.
    ***Note:*** The relationship between the F1 scores and SNRs of both NOS1 and HIF1A staining are shown in Figure 8B. The F1 scores (x-axis) were calculated using the default Nikon IoU threshold of 0.5 and plotted against the corresponding average SNR (y-axis). NOS1 staining with F1 ≥ 0.5 had SNRs ≥ ∼19, while HIF1A achieved F1 ≥ 0.5 at SNRs as low as ∼4. Images below these thresholds should be excluded from AI-based segmentation to ensure reliable performance.
33. AI neuron volume segmentation:
  a. Use the built-in GA3 recipe system to create a new GA3 recipe (see Figure 5B for the expected final recipe layout). Apply background reduction using the nodes DarkSpots, MeanIntensity, ModifyColumns, and SubtractConstant.
  b. Use the search tab in the top-right corner of the GA3 recipe window to locate these nodes and drag one of each onto the programming window.
    i. Click and drag the DarkSpots’s “A” module to the DAPI channel.
    ii. Set the value of “Typical Diameter” to 10 μm.
    iii. Set “Contrast” to 0.010, “Z-axis elongation” to 1:1.
    iv. Set the threshold range should go from a minimum of 0 to a maximum of 1.
    v. Set “Grow” to disabled.
    vi. Set “Output” to “Circular Area.”
    vii. Click and drag the MeanIntensity’s “A” module to the DAPI channel.
    viii. Click and drag the “B” module to the colored node attached to DarkSpots’s binary layer node (see Figure 5A).
      ***Note:*** This creates a segmentation layer that appears as a colored node in GA3.
    ix. Click and drag the 'A' module of the MeanIntensity node to the DAPI channel.
    x. Then, click and drag the 'B' module to the colored node created by DrawRectangle.
    xi. Click and drag the 'A' module of the ModifyColumns node to the Records node connected to MeanIntensity.
    xii. Open the drop-down menu in ModifyColumns and uncheck the box next to 'ZStackIndex' (see Figure 5B).
    xiii. Click and drag the 'A' module of the SubtractConstant node to the DAPI channel.
    xiv. Right-click on SubtractConstant and enable the P0 variable to create a P0 module connection.
    xv. Attach the P0 module to the Records node connected to ModifyColumns.
      ***Note:*** This will create a new DAPI channel node. Repeat steps b through r for each channel (HIF1A and NOS1).
    xvi. Apply the Segment.ai node directly to the newly created NOS1 channel.
    xvii. Click on the drop-down menu and set the AI software to the corresponding model trained previously.
      ***Note:*** It is recommended to use the NIS AI software for segmentation (Segment_ai), as it significantly reduces the time required for segmentation and quantification. Threshold segmentation (plus cleaning up any unnecessary segmentations) may take up to 45 min per image stack, whereas AI segmentation can complete this step in as little as 10 sec. The Nikon GA3 recipe used for this quantification, as shown in Figure 5, has been deposited in Zenodo and is publicly available at https://doi.org/10.5281/zenodo.15077205. A step-by-step tutorial video (Methods Video 1) demonstrating the use of this GA3 recipe is provided as supplemental material and is also available at Zenodo (https://doi.org/10.5281/zenodo.15064787). A step-by-step tutorial video demonstrating the use of this GA3 recipe is provided as supplemental material and is also available at Zenodo (https://doi.org/10.5281/zenodo.15064787).
    xviii. Create a ConnectCells node.
    xix. Click and drag the ConnectCells node’s “A” module onto the AI segmentation node to merge the 2D segments into a complete 3D segmentation object.
    xx. Create a Volume node.
    xxi. Click and drag the Volume node’s “A” module to the ConnectCells segmentation node.
    xxii. Add a ModifyColumns node for both HIF1A and NOS1.
    xxi. Click and drag the ModifyColumns node’s “A” module to the Volume’s Records node.
    xxii. Click on the drop-down menu and remove the Entity column by unselecting the checkbox.
    xxiii. Create an ExportCSV node to export the table as a CSV.
      ***Note:*** ConnectCells combines the 2D segmentation layers into a single 3D segmentation layer, generating 3D objects for analysis. Volume measures each individual 3D object and outputs the data as a table. ModifyColumns is used again to remove unnecessary columns generated by the Volume node. To ensure accurate volume measurements, we excluded neurons that were partially clipped at the edges of the z stack. ExportCSV converts the table into a CSV file.
      **CRITICAL:** Proper segmentation is crucial for accurate measurements. Threshold segmentation may require manual editing to remove excess areas, which can introduce variability. AI training and segmentation, however, can eliminate the need for these manual adjustments, providing more accurate and consistent results while minimizing personnel bias.

**Table 5:** Summary of key steps in fluorescence intensity quantification and neuron volume segmentation

Process	Step	Description
Background reduction for sum fluorescence quantification	31 d-e, 31 f-l, 31 m	Identify and remove background fluorescence to enhance signal detection.
Fluorescence intensity quantification	31 n-s	Compute sum intensity of fluorescent signals for markers of interest using AI segmented areas as regions of interest.
Estimation of quality	32 a-c	Assess AI performance, connect ‘QualityEstimate_ai’ to NOS1 and HIF1A binary layers to determine signal-to-noise ratio.
Validation of AI-supported segmentation results (F1 score)	32 e-h	Upload manual segmentation, run SegmentationAccuracy, compile with AppendColumns, and export results to Excel.
Background reduction for volume	33 a	Identify and remove background fluorescence using DarkSpots for AI-based volume quantification.
Neuron volume segmentation	33 b	Create AI-segmented binary layers on identified neurons using fluorescent signals of markers of interest. Combine z stack images to construct three-dimensional neuron representations and measure their volume.

Methods Video S1. Step-by-step tutorial demonstrating the use of the GA3 recipe for AI-supported fluorescence quantification, related to Step 31

Methods Video S2. Z-stack comparison of AI and manual segmentation of NOS1-positive enteric neurons, related to Step 32 This video compares AI-segmented (yellow) and manually segmented (white) NOS1+ enteric neurons across a confocal Z-stack. Scale bar: 50 μm.

## Expected outcomes

This protocol enables accurate identification, segmentation, and quantification of NOS1-positive enteric neurons in the human gastric tunica muscularis. By integrating immunostaining with AI-based image analysis, it provides precise data on the colocalization of target proteins within enteric neurons, improving our understanding of their role in gastrointestinal motility in both health and disease. The AI-assisted workflow ensures reproducibility, minimizes manual errors, and offers an unbiased approach to data analysis. Additionally, the use of AI training optimizes neuron segmentation, producing high-quality and consistent results that enhance the reliability of downstream analyses. Despite being reproducible, these analyses should be ideally supplemented with additional methods, such as flow cytometry and fluorescence-activated cell sorting, to validate and confirm the experimental results.^7^^,^^9^^,^^10^

## Limitations

The protocol’s effectiveness depends on the quality of tissue preparation, immunostaining, and imaging. While AI-assisted segmentation reduces manual bias, it is susceptible to errors when training images are suboptimal, or new images exhibit significantly different morphologies. These challenges may be particularly pronounced when working with transgenic models or diseased tissues. Despite extensive training, the AI software may still misclassify some cells, and achieving perfect segmentation accuracy remains unattainable. Additionally, variations in antibody performance and imaging conditions may further impact reproducibility and data interpretation. To ensure reliable performance, users should compare AI-generated results with their own manually annotated datasets, particularly when adapting the model to specific experimental contexts. A separate limitation involves the use of 3D image analysis, which is not directly compatible with common validation methods that are restricted to 2D. To confirm the accuracy of results, complementary approaches such as quantitative polymerase chain reaction, western immunoblotting on fluorescence-activated cell sorting-sorted cells, or flow cytometry should be considered.^9^^,^^10^^,^^11^ Metrics such as the F1 score and percentage cell-count accuracy, derived from comparisons against manually annotated datasets, can offer objective and reproducible measures of model performance as shown in Figure 8B. Another limitation is that the Nikon NIS-Elements software used for AI-based image analysis is a commercial tool that typically requires access to a Nikon microscope system. As alternatives for researchers without access to this software, several open-source tools can perform similar AI-driven image analysis can be considered. These include StarDist, which detects round-shaped cells like enteric neurons using deep learning, deepImageJ, which enables deep learning-based segmentation within ImageJ/Fiji, Cellpose, a generalist cell segmentation tool with pre-trained models, and QuPath, an open-source platform for whole-slide image analysis, including cell classification and segmentation.^12^ Incorporating these alternatives expands accessibility and allows researchers to apply AI-driven segmentation across different imaging systems.

## Troubleshooting

### Problem 1

NOS1-positive enteric neurons are poorly defined (related to Step: preparing cryosections of human gastric muscles).

### Potential solution

Process the tissues promptly after gastric sleeve surgery to preserve their quality, as the issue may be related to compromised tissue quality.

### Problem 2

The signaling of HIF1A is weak (related to Step: Staining of NOS1 and HIF1A for quantification).

### Potential solution

To prevent degradation of HIF1A antibody quality and reduced binding efficiency, minimize repeated freeze-thaw cycles by aliquoting antibodies into small volumes. This avoids the need for repeated freezing and thawing.

Additionally, optimize permeabilization steps to enhance antibody penetration, accounting for the varying thickness of the human gastric tunica muscularis.

### Problem 3

The AI software is segmenting a significant number of false-positive cells (related to Step: AI neuron volume segmentation).

### Potential solution

Review the training images for potential errors, as the AI software’s accuracy depends on the quality of the data it was trained on. Increase the diversity of training images by including examples with varying cell densities and morphologies, such as sparsely distributed cells, densely packed cells, and atypically shaped cells. This approach can help the AI software adapt to inconsistencies in cell appearance, improving its segmentation accuracy.

### Problem 4

The AI software training program is not working. The source channels, binary layer options, or another setting appears highlighted in red (related to Step: AI Training for neuron segmentation).

### Potential solution

This issue is often caused by inconsistencies in the source channels or binary layers, such as mismatched or differently named binary layers. Double-check that the binary layer names and source channels are consistent across all images. Inconsistencies will prevent the training program from functioning. To resolve the problem quickly, identify and remove any files with discrepancies.

### Problem 5

The AI software is not segmenting the images well, and some positive cells are missed (related to Step: Immunohistochemistry analysis and quantification).

### Potential solution

The AI software’s performance depends on the quality of the images used for training. Ensure you are using high-quality images with accurate segmentation. Consider increasing the number of slices taken from each image to improve fidelity and enhance the AI software’s ability to map segmentation to each pixel of fluorescence. Avoid artificially increasing the number of training images by including subpar-quality images, as this may confuse the AI software and lead to more errors. Ensure training images are high quality and accurately segmented. Increase image slices for better fidelity and avoid using low-quality images to maintain accuracy.

## Resource availability

### Lead contact

Further information and requests for resources and reagents should be directed to and will be fulfilled by the lead contact, Yujiro Hayashi (hayashi.yujiro@mayo.edu).

### Technical contact

Technical questions regarding the execution of this protocol should be directed to and will be answered by the technical contact, Egan L. Choi (choiegan00@gmail.com).

### Materials availability

This study did not generate any new unique reagents.

### Data and code availability

This study does not report any original code. Two datasets generated during the study have been deposited to Zenodo, an open-access research repository that provides DOI-based citation and long-term preservation. The first is the GA3 recipe used for AI-supported image analysis (https://doi.org/10.5281/zenodo.15077205). The second is a tutorial video demonstrating the use of the GA3 recipe and comparing AI-supported versus manual segmentation of NOS1^+^ enteric neurons across a confocal z stack (https://doi.org/10.5281/zenodo.15064787). This video is also included as supplementary material. The graphical abstract and figure thumbnails for this study were prepared using BioRender.com.

## Acknowledgments

This study was supported in part by National Institutes of Health, National Institute of Diabetes and Digestive and Kidney Diseases grants R01 DK121766, R01 DK126827, R01 DK131455, P01 DK068055, and P30 DK084567 (Epigenomics and Spatial Biology Core), and the Mayo Clinic Center for Individualized Medicine. The funding agencies had no role in the study analysis or writing of the manuscript. Its contents are solely the responsibility of the authors. The authors are grateful to Dr. Todd A. Kellogg, Dr. Michael L. Kendrick, Dr. Travis J. McKenzie, and Dr. Omar Ghanem for the gastric sleeve samples. We would like to thank Mr. Eugene W. Krueger, manager of the Microscopy and Microfluidics Core (directed by Dr. Mark McNiven and funded by NIH P30 DK084567), for his technical support. The funding agencies had no role in the study analysis or writing of the manuscript. Its contents are solely the responsibility of the authors.

## Author contributions

Conceptualization, E.L.C., Y.H., and T.O.; investigation, E.L.C., Y.Z., N.M.H., and Y.H.; writing – original draft, Y.H.; writing – review and editing, E.L.C., Y.Z., F.G., N.M.H., A.S.Q., A.E.B., Y.H., and T.O.; visualization, E.L.C., N.M.H., and Y.H.; resources, A.E.B.; supervision, Y.H. and T.O. E.L.C. and Y.Z. contribute equally.

## Declaration of interests

The authors declare no competing interests.

## Declaration of generative AI and AI-assisted technologies in the writing process

During the preparation of this manuscript, the authors utilized OpenAI’s ChatGPT for assistance in revising and refining the text. Following the use of these tools, the authors thoroughly reviewed and edited the content as necessary and take full responsibility for the integrity and accuracy of the final publication.

## References

[1] Gao F., Hayashi Y., Saravanaperumal S.A., Gajdos G.B., Syed S.A., Bhagwate A.V., Ye Z., Zhong J., Zhang Y., Choi E.L. (2023). Hypoxia-Inducible Factor 1alpha Stabilization Restores Epigenetic Control of Nitric Oxide Synthase 1 Expression and Reverses Gastroparesis in Female Diabetic Mice. Gastroenterology.

[2] Rao M., Gershon M.D. (2016). The bowel and beyond: the enteric nervous system in neurological disorders. Nat. Rev. Gastroenterol. Hepatol..

[3] Watkins C.C., Sawa A., Jaffrey S., Blackshaw S., Barrow R.K., Snyder S.H., Ferris C.D. (2000). Insulin restores neuronal nitric oxide synthase expression and function that is lost in diabetic gastropathy. J. Clin. Investig..

[4] Zinchuk V., Zinchuk O., Okada T. (2007). Quantitative colocalization analysis of multicolor confocal immunofluorescence microscopy images: pushing pixels to explore biological phenomena. Acta Histochem. Cytochem..

[5] Taheri N., Choi E.L., Nguyen V.T.T., Zhang Y., Huynh N.M., Kellogg T.A., van Wijnen A.J., Ordog T., Hayashi Y. (2024). Inhibition of EZH2 Reduces Aging-Related Decline in Interstitial Cells of Cajal of the Mouse Stomach. Cell. Mol. Gastroenterol. Hepatol..

[6] Choi E.L., Taheri N., Hayashi Y. (2025). Protocol for AI-based segmentation and quantification of interstitial cells of Cajal in murine gastric muscle. STAR Protoc..

[7] Izbeki F., Asuzu D.T., Lorincz A., Bardsley M.R., Popko L.N., Choi K.M., Young D.L., Hayashi Y., Linden D.R., Kuro-o M. (2010). Loss of Kitlow progenitors, reduced stem cell factor and high oxidative stress underlie gastric dysfunction in progeric mice. J. Physiol..

[8] Grover M., Farrugia G., Lurken M.S., Bernard C.E., Faussone-Pellegrini M.S., Smyrk T.C., Parkman H.P., Abell T.L., Snape W.J., Hasler W.L. (2011). Cellular changes in diabetic and idiopathic gastroparesis. Gastroenterology.

[9] Hayashi Y., Bardsley M.R., Toyomasu Y., Milosavljevic S., Gajdos G.B., Choi K.M., Reid-Lombardo K.M., Kendrick M.L., Bingener-Casey J., Tang C.M. (2015). Platelet-Derived Growth Factor Receptor-alpha Regulates Proliferation of Gastrointestinal Stromal Tumor Cells With Mutations in KIT by Stabilizing ETV1. Gastroenterology.

[10] Schwamb B., Pick R., Fernández S.B.M., Völp K., Heering J., Dötsch V., Bösser S., Jung J., Beinoraviciute-Kellner R., Wesely J. (2015). FAM96A is a novel pro-apoptotic tumor suppressor in gastrointestinal stromal tumors. Int. J. Cancer.

[11] Wang Y., Marino-Enriquez A., Bennett R.R., Zhu M., Shen Y., Eilers G., Lee J.C., Henze J., Fletcher B.S., Gu Z. (2014). Dystrophin is a tumor suppressor in human cancers with myogenic programs. Nat. Genet..

[12] Sorensen L., Humenick A., Poon S.S.B., Han M.N., Mahdavian N.S., Rowe M.C., Hamnett R., Gómez-de-Mariscal E., Neckel P.H., Saito A. (2024). Gut Analysis Toolbox - automating quantitative analysis of enteric neurons. J. Cell Sci..

